# Going to sleep in the supine position is a modifiable risk factor for late pregnancy stillbirth; Findings from the New Zealand multicentre stillbirth case-control study

**DOI:** 10.1371/journal.pone.0179396

**Published:** 2017-06-13

**Authors:** Lesley M. E. McCowan, John M. D. Thompson, Robin S. Cronin, Minglan Li, Tomasina Stacey, Peter R. Stone, Beverley A. Lawton, Alec J. Ekeroma, Edwin A. Mitchell

**Affiliations:** 1 Department of Obstetrics and Gynaecology, University of Auckland, Auckland, New Zealand; 2 Department of Paediatrics and Child Health, University of Auckland, Auckland, New Zealand; 3 School of Healthcare, University of Leeds, Leeds, United Kingdom; 4 Women’s Health Research Centre, University of Otago, Wellington, New Zealand; Universitat de Barcelona, SPAIN

## Abstract

**Objective:**

Our objective was to test the primary hypothesis that maternal non-left, in particular supine going-to-sleep position, would be a risk factor for late stillbirth (≥28 weeks of gestation).

**Methods:**

A multicentre case-control study was conducted in seven New Zealand health regions, between February 2012 and December 2015.

Cases (n = 164) were women with singleton pregnancies and late stillbirth, without congenital abnormality. Controls (n = 569) were women with on-going singleton pregnancies, randomly selected and frequency matched for health region and gestation.

The primary outcome was adjusted odds of late stillbirth associated with self-reported going-to-sleep position, on the last night. The last night was the night before the late stillbirth was thought to have occurred or the night before interview for controls. Going-to-sleep position on the last night was categorised as: supine, left-side, right-side, propped or restless. Multivariable logistic regression adjusted for known confounders.

**Results:**

Supine going-to-sleep position on the last night was associated with increased late stillbirth risk (adjusted odds ratios (aOR) 3.67, 95% confidence interval (CI) 1.74 to 7.78) with a population attributable risk of 9.4%. Other independent risk factors for late stillbirth (aOR, 95% CI) were: BMI (1.04, 1.01 to 1.08) per unit, maternal age ≥40 (2.88, 1.31 to 6.32), birthweight <10^th^ customised centile (2.76, 1.59 to 4.80), and <6 hours sleep on the last night (1.81, 1.14 to 2.88). The risk associated with supine-going-to-sleep position was greater for term (aOR 10.26, 3.00 to 35.04) than preterm stillbirths (aOR 3.12, 0.97 to 10.05).

**Conclusions:**

Supine going-to-sleep position is associated with a 3.7 fold increase in overall late stillbirth risk, independent of other common risk factors. A public health campaign encouraging women not to go-to-sleep supine in the third trimester has potential to reduce late stillbirth by approximately 9%.

## Introduction

Stillbirth remains one of few potentially avoidable maternal and child health problems and is now approximately twice as common as neonatal death [1, 2]. The 2016 Lancet “Ending Preventable Stillbirth Series” highlighted differences in rates of late stillbirth (at ≥28 weeks’) between high-income countries ranging from #1.3/1000 to 8.8/1000 births [2]. Disparities also exist between ethnic groups within high-income countries [1, 3]. Such variations suggest it should be possible to further reduce late stillbirth rates in many settings. Accordingly there is an urgent need to identify simple and cost effective preventative interventions for stillbirth [4]. Furthermore at least 30% of late stillbirths are currently classified as unexplained [1, 2]. Although a number of studies have examined risk factors for stillbirth, many have only been able to use routinely collected data [5, 6] or have been systematic reviews [7] and therefore have been unable to explore detailed relationships with maternal lifestyle and personal habits.

Approximately one third of life is spent asleep, and until recently there has been little research on the impact of maternal sleep practices on the fetus. Our Auckland Stillbirth Study was the first to report an association between going-to-sleep position and late stillbirth risk, with mothers who did not settle to sleep on their left side, the night before the baby was suspected to have died, having a two-fold increase in risk [8]. The highest risk, among non-left sided sleepers, occurred in women who went to sleep supine aOR 2.54, 95% CI (1.04 to 6.18). The relationship between supine going-to-sleep position and late stillbirth is biologically plausible as supine position in late pregnancy is associated with pathophysiological effects with potential to compromise fetal wellbeing. These include reduced: maternal cardiac output [9], uterine blood flow [10], and pulsatility index in the fetal middle cerebral artery (a surrogate for fetal hypoxia) [11]. Supine sleep position is also associated with sleep disturbed breathing and obstructive sleep apnea, which have each been associated with pregnancy complications [12, 13].

In the Auckland Stillbirth Study we also found that women who regularly slept during the day (aOR 2.04, 95% CI (1.26 to 3.30) and those with long (>8 hours) night time sleep duration (aOR 1.71, 95% CI (0.99 to 2.95) also had an increased risk of late stillbirth. However, it is not clear whether these associations are causal or incidental.

We now report results from the New Zealand multicentre stillbirth study in which we tested the primary hypothesis that non-left, and specifically supine going-to-sleep position, would be associated with increased risk of late stillbirth. In addition we also tested the hypothesis that increased sleep duration and sleeping during the day would be associated with increased late stillbirth risk.

## Methods

The detailed study protocol is accessible on figshare doi: 10.17608/k6.auckland.3483134.v1 [14].

### Study design and population

This multicentre case-control study was conducted in seven of twenty New Zealand health regions (District Health Boards, DHB). Criteria for choosing these regions, was that they had the largest numbers of late stillbirths in New Zealand between 2007 and 2010 [14]. Two thirds of all New Zealand births occur in these seven health regions (Waitemata, Auckland, Counties Manukau, Waikato, MidCentral, Capital & Coast, and Canterbury). Recruitment occurred between February 2012 and December 2015. Ethical approval was obtained from the Northern “X” Regional Ethics Committee: NTX/06/05/054.

Women with multiple pregnancies and babies identified with major congenital abnormalities at any stage of the study were excluded. Cases were consenting women with a stillbirth at ≥28 weeks of gestation. We obtained the distribution of stillbirths by health region and gestation using New Zealand stillbirth data over a three-year period (2007–9) [15]. Controls were women with ongoing pregnancies randomly selected with frequency matching to a projected distribution of stillbirths, ensuring that controls would be at a similar gestation to cases in each participating health region. We planned to select two controls for each estimated eligible case in each health region.

### Procedures and variables

Eligible subjects were given a brief description of the study by their midwife or doctor and asked whether the research midwife could contact them to explain the study and invite them to participate. If the woman agreed, a time and place for interview was arranged and written consent was obtained. Eligible participants were informed that the broad aim of the study was to identify modifiable risk factors for late stillbirth and specific hypotheses were not discussed. If the woman did not consent, age, ethnicity and parity were collected without identifiable information. Interpreters were organised for women who had difficulty reading or speaking in English.

Data were obtained by structured face-to-face interview (S1 Appendix) conducted by research midwives employed part-time in each recruitment centre and from medical records. For cases the interview occurred as soon after stillbirth as possible and for controls, as close to the allocated gestation as possible. Data collected in the interview included: demographic, lifestyle, general health and antenatal care details and information about a range of sleep practices [14]. Data about sleep practices were collected at the end of the interview. Height was measured by the research midwife and body mass index calculated using the earliest weight recorded in pregnancy. A single prioritized maternal ethnicity was determined as recommended by the New Zealand Ministry of Health [16]. Social deprivation was derived from the address where the participant lived during pregnancy, with category one least deprived and category five most deprived [17].

The primary outcome was the odds of late stillbirth associated with maternal self-reported going-to-sleep position, on the last night. The last night was the night before late stillbirth was thought to have occurred or the night before interview for controls. Information about usual going-to-sleep position in the last week was also collected. Going-to-sleep position on the last night was classified as: left side, right side, restless, supine (lying on the back), on the front, or propped. Restless going-to-sleep referred to women who reported that they had changed position frequently when settling to sleep and could not recall the position they finally fell asleep in. In women who did not remember any details of their going-to-sleep position, the position was recorded as unknown. Usual going-to-sleep position in the last week was classified as: left side, right side, variable side, supine (lying on the back), on the front or propped. To assist the participant to recall her going-to-sleep position the woman was asked to visualise herself lying in bed.

Participants were also asked if they got up at night to the toilet and how many hours they thought they had slept at night on the last night. Sleep duration was categorised as: <6 hours; 6 to 8 hours; and >8 hours, as per our previous study [8]. The frequency of sleeping during the daytime in the last week was also recorded.

Customised birthweight centiles, that adjust for maternal characteristics (height, early pregnancy weight, ethnicity and parity) as well as gestation at delivery and infant sex, were calculated for all infants [18]. The gestation used to calculate the centile for cases was the day the mother thought the baby had died and for controls the gestation at birth. Small for gestational age (SGA) was defined as birthweight <10^th^ customised centile [18].

All stillbirths were classified using the Perinatal Society of Australia and New Zealand Classification system [19]. Deaths classified as due to fetal growth restriction require either antenatal diagnosis of fetal growth restriction with abnormal Doppler studies and or specified placental pathology. If growth restriction was not identified before birth, late stillbirths with a customised birthweight centile <10^th^ are typically classified as unexplained [19].

### Statistical analysis

We estimated that over a three year recruitment period there would be 415 eligible cases, 291 of whom would participate [15], based on 70% recruitment in The Auckland Stillbirth Study [8], and 830 eligible controls (estimated 582 participants). Given this sample size, a significance level of 0.05, power of 0.8 and prevalence of a risk factor in controls of between 30% and 60% (prevalence of non-left sleep position in The Auckland Stillbirth Study was 57%) we could detect an odds ratio (OR) of 1.50. With a 5% prevalence of a risk factor (supine sleep position in The Auckland Stillbirth Study controls) we could detect an OR of 2.2.

Statistical analyses were performed using SAS version 9.6 (SAS Institute Inc., Cary NC USA). Generalized additive models were fitted to explore the relationship between continuous variables (maternal age, BMI, birthweight centile) and stillbirth, and determine whether variables should be analysed continuously or categorically.

Differences between categorical variables were tested by chi-square tests. Continuous variables were compared using Wilcoxon Rank Sum tests. Univariable analysis was performed to evaluate the association between sleep practices and late stillbirth risk. A multivariable model was developed incorporating ethnicity and deprivation index, variables associated with increased risk of stillbirth based on previous literature (age, BMI, parity, smoking, SGA status), other sleep variables significant in univariable analysis, and variables used to select cases and controls (gestation and DHB). Unconditional logistic regression was used to adjust for potential confounders. No imputation was performed for missing data. Women who could not recall their going-to-sleep position on the last night (unknown going-to- sleep position) were excluded from the multivariable model. We also carried out a stratified analysis by term (≥37 weeks’) and preterm (≥28 to 36 weeks’) gestation. The c statistic has been calculated to assess model performance.

Statistical significance was defined at the 5% level. OR and adjusted odds ratios (aOR) with 95% confidence intervals (CI) were used to estimate risk. Population Attributable Risk (PAR) was calculated using unadjusted OR for the primary outcome, supine sleep position, and other potentially modifiable risk factors that remained significant in multivariable analysis [20].

## Results

During recruitment there was a 40% national reduction in late stillbirths, compared with estimated numbers based on data from 2007–9 [15]. Fewer cases were therefore recruited, 164 vs 291 projected, whereas numbers of controls were similar to projected, 569 vs 582. The final ratio of cases to controls was thus 1: 3.5. The rate of recruitment was 65.9% for cases and 62.2% for controls (Table 1) and recruitment for cases and controls occurred contemporaneously throughout the study.

**Table 1:** Comparison of basic demographic characteristics between eligible non-participants and women who consented to participate in the study.

	Cases			Controls		
Characteristic	Participants (n = 164)	Non-participants (n = 85)	P value	Participants (n = 569)	Non-participants (n = 346)	P value
Age (years)						
<20	9 (5.5)	3 (3.5)	P = 0.58	17 (3.0)	18 (5.2)	P = 0.19
20–39	141 (86.0)	77 (90.6)		532 (93.5)	313 (90.5)	
≥40	14 (8.5)	5 (5.9)		20 (3.5)	15 (4.3)	
Ethnicity						
Māori	26 (16.0)	22 (25.9)	P = 0.40	58 (10.2)	51 (14.7)	P = 0.01
Pacific	38 (23.2)	20 (23.5)		86 (15.1)	50 (14.5)	
Indian	17 (10.4)	10 (11.8)		77 (13.5)	23 (6.7)	
Other Asian	13 (7.9)	6 (7.1)		72 (12.7)	46 (13.3)	
European	65 (39.6)	26 (30.6)		263 (46.2)	162 (46.8)	
Other	5 (3.1)	1 (1.2)		13 (2.3)	14 (4.0)	
Parity						
0	76 (46.3)	31 (36.5)	P = 0.02	245 (43.1)	141 (40.7)	P = 0.05
1–3	80 (48.8)	42 (49.4)		308 (54.1)	184 (53.2)	
≥4	8 (4.9)	12 (14.1)		16 (2.8)	21 (6.1)	

Data are presented as number (percentage)

Baseline demographic characteristics were compared between eligible non-participants and recruited participants (Table 1). Women of high parity were underrepresented in cases and controls. Indian women were over-represented and Māori under-represented in participating compared with eligible controls. To further check representativeness of the recruited population, we compared the univariable odds for late stillbirth for age, ethnicity and parity, from the eligible cases and controls (participants and non-participants) with the odds ratios from recruited cases and controls, and there were no significant differences (results not shown).

Classification of most likely cause of late stillbirth [19] demonstrated that 38.4% of all stillbirths and 44.8% of stillbirths after 37 weeks’ were unexplained (Table 2).

**Table 2:** Cause of late stillbirth using Perinatal Society of Australia and New Zealand death classification system (PSANZ-PDC) in preterm and term cases.

	Total cases (n = 164)	Preterm cases (28–36 weeks’) (n = 68, 41.5%)	Term cases ≥37 weeks’ (n = 96, 58.5%)
Unexplained	58 (35.4)	20 (29.4)	38 (39.6)
Perinatal infection	5 (3.0)	2 (2.9)	3 (3.1)
Hypertension	8 (4.9)	4 (5.9)	4 (4.2)
Haemorrhage	13 (7.9)	9 (13.2)	4 (4.2)
Maternal conditions	12 (7.3)	10 (14.7)	2 (2.1)
Specific perinatal condition	25 (15.2)	10 (14.7)	15 (15.6)
Hypoxic peripartum death	14 (8.5)	0 (0)	14 (14.6)
Fetal growth restriction	28 (17.2)	12 (17.7)	16 (16.7)
Spontaneous preterm	1 (0.6)	1 (1.5)	0 (0)

Data are presented as number (percentage)

**Fig 1:**
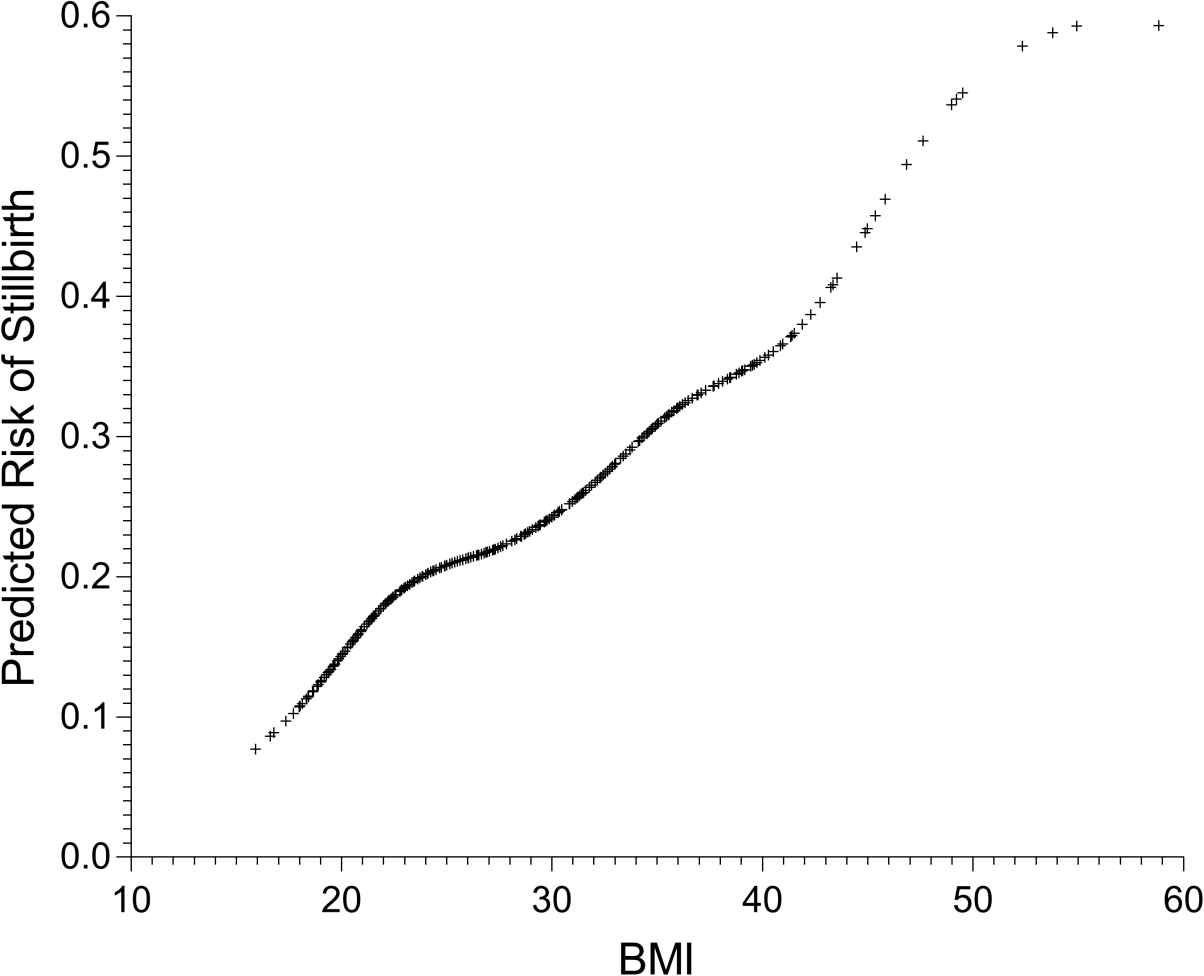
Relationship between BMI and late stillbirth risk.

Median (interquartile range, IQR) gestation at the estimated time of late stillbirth was 37.7 (34.1 to 39.9) weeks’ and at interview for controls was 37.4 (34.0 to 38.9) weeks’ (p = 0.003) and 97 (58.5%) of stillbirths occurred at ≥37 weeks’. The median (IQR) time to interview after the estimated date of stillbirth was 24 (16 to 32) days. Pregnancy weight was measured before 20 weeks of gestation in 91% of women and a linear relationship between increasing BMI and late stillbirth risk was demonstrated (Fig 1).

On the last night, there was an increase in late stillbirth risk in univariable analysis in women who went to sleep supine (OR 3.63, 95% CI 1.87 to 7.04) and in those who reported they were restless going-to-sleep (OR 2.68, 95% CI 1.31 to 5.47) (Table 3). On the last night before late stillbirth there was also a borderline increase in risk with non-left going-to-sleep positions (OR 1.42, 95% CI 1.00 to 2.02) (Table 3).

**Table 3:** Demographic characteristics and sleep factors among 164 women who experienced a late stillbirth, between 2012 and 2015, compared to 569 controls.

Characteristic	Cases (n = 164)	Controls (n = 569)	Unadjusted odds ratio (95% CI)	Adjusted odds ratio (95% CI)
Age (years)				
<20	9 (5.5)	17 (3.0)	2.00 (0.87 to 4.58)	1.28 (0.46 to 3.59)
20–39	141 (86.0)	532 (93.5)	1.00	1.00
≥40	14 (8.5)	20 (3.5)	2.64 (1.30 to 5.36)	2.88 (1.31 to 6.32)
Ethnicity				
Māori	26 (15.9)	58 (10.2)	1.81 (1.06 to 3.10)	1.20 (0.60 to 2.41)
Pacific	38 (23.2)	86 (15.1)	1.79 (1.12 to 2.86)	1.22 (0.62 to 2.41)
Indian	17 (10.4)	77 (13.5)	0.89 (0.50 to 1.61)	1.01 (0.48 to 2.13)
Other Asian	13 (7.9)	72 (12.7)	0.73 (0.38 to 1.40)	1.05 (0.50 to 2.20)
European	65 (39.6)	263 (46.2)	1.56 (0.54 to 4.52)	1.00
Other	5 (3.1)	13 (2.3)	1.81 (1.06 to 3.10)	1.56 (0.48 to 5.12)
Parity				
0	76 (46.3)	245 (43.1)	1.19 (0.84 to 1.71)	1.25 (0.83 to 1.89)
1–3	80 (48.8)	308 (54.1)	1.00	1.00
≥4	8 (4.9)	16 (2.8)	1.93 (0.80 to 4.66)	0.96 (0.35 to 2.64)
Social deprivation level				
1–2	40 (24.4)	207 (36.4)	1.00	1.00
3	34 (20.7)	109 (19.2)	1.61 (0.97 to 2.70)	1.45 (0.82 to 2.59)
4–5	90 (54.9)	253 (44.5)	1.84 (1.22 to 2.79)	1.39 (0.81 to 2.38)
Earliest pregnancy BMI				
	26.6 (23.2 to 33.5)	24.8 (22.1 to 29.5)	1.06 (1.03 to 1.09)	1.04 (1.01 to 1.08)
Marital status				
Married	91 (55.5)	389 (68.4)	1.00	1.00
Co-habiting	53 (32.3)	151 (26.5)	1.50 (1.02 to 2.21)	1.29 (0.80 to 2.10)
Single	20 (12.2)	29 (5.1)	2.95 (1.60 to 5.41)	1.46 (0.65 to 3.27)
Smoking in pregnancy				
Yes	37 (22.6)	66 (11.6)	2.22 (1.42 to 3.47)	1.20 (0.66 to 2.15)
No	127 (77.4)	503 (88.4)	1.00	1.00
Baby birthweight centile				
<10	47 (28.7)	73 (12.8)	3.56 (2.17 to 5.82)	2.76 (1.59 to 4.80)
10–49.99	62 (37.8)	219 (38.5)	1.56 (1.01 to 2.41)	1.48 (0.92 to 2.38)
50–89.99	42 (25.6)	232 (40.8)	1.00	1.00
>90	13 (7.9)	45 (7.9)	1.6 (0.79 to 3.21)	1.36 (0.63 to 2.95)
Hours of night time sleep on the last night				
<6 hours	53 (32.2)	106 (18.6)	2.01 (1.34 to 3.00)	1.81 (1.14 to 2.88)
6–8 hours	89 (54.2)	357 (62.7)	1.00	1.00
>8 hours	22 (13.4)	106 (18.6)	0.83 (0.50 to 1.39)	0.79 (0.44 to 1.41)
Getting up to toilet during the last night				
0	35 (21.3)	92 (16.2)	1.41 (0.91 to 2.17)	1.55 (0.93 to 2.56)
≥ 1	129 (78.7)	477 (83.8)	1.00	1.00
Sleep during the daytime in the last week				
Never	34 (20.7)	113 (19.8)	1.00	1.00
Occasionally	21 (12.8)	88 (15.5)	0.79 (0.43 to 1.46)	0.85 (0.42 to 1.69)
1–2 times	42 (25.6)	162 (28.5)	0.86 (0.52 to 1.44)	0.91 (0.51 to 1.61)
3–4 times	23 (14.0)	108 (18.9)	0.71 (0.39 to 1.28)	0.62 (0.32 to 1.23)
≥ 5 times	44 (26.8)	98 (17.2)	1.49 (0.89 to 2.52)	1.27 (0.70 to 2.33)
Going-to-sleep position on the last night *				
Left side	78 (47.6)	328 (57.6)	1.00	1.00
Non-Left	81 (49.4)	240 (42.2)	1.42 (1.00 to 2.02)	
Right side	44 (26.8)	187 (32.9)	0.99 (0.66 to 1.49)	0.92 (0.58 to 1.44)
Restless	14 (8.5)	22 (3.9)	2.68 (1.31 to 5.47)	1.98 (0.87 to 4.50)
Supine	19 (11.6)	22 (3.9)	3.63 (1.87 to 7.04)	3.67 (1.74 to 7.78)
Propped	4 (2.4)	9 (1.6)	1.87 (0.56 to 6.23)	1.11 (0.30 to 4.06)
Validation model using going-to-sleep position in the last week				
Going-to-sleep position in the last week ^#^				
Left side	85 (51.8)	302 (53.1)	1.00	–
Non-Left	78 (47.6)	266 (46.7)	1.04 (0.74 to 1.48)	
Right side	42 (25.6)	171 (30.1)	0.87 (0.58 to 1.32)	0.82 (0.52 to 1.30)^†^
Variable side	15 (9.2)	72 (12.7)	0.74 (0.40 to 1.36)	0.85 (0.44 to 1.65)^†^
Supine	15 (9.2)	16 (2.8)	3.33 (1.58 to 7.01)	3.46 (1.49 to 8.03)^†^
Propped	5 (3.1)	7 (1.2)	2.54 (0.79 to 8.20)	2.10 (0.57 to 7.71)^†^
On front	1 (0.6)	0	NA	NA

Data are presented as number (percentage) or median (IQR). Two multivariable models have been conducted using either last night or last week going-to-sleep position. The adjusted odds ratio (aOR) for other variables are from the multivariable model using last night going-to sleep position. Multivariable models are adjusted for matching terms (gestation at interview in controls and at diagnosis of stillbirth for cases and district health board), and all the other variables in the table. No imputation for missing data.

* In five cases and one control sleep position on the last night was unknown and they were excluded from the multivariable model

^#^ One case and one control did not recall their sleep position in the last week and were excluded from the multivariable model

^†^ Adjusted odds ratio (aOR) using last week going-to-sleep position.

Risk factors for late stillbirth significant in multivariable analysis were: supine going-to-sleep position on the last night (aOR 3.67, 95% CI 1.74 to 7.78), <6 hours sleep on the last night (aOR 1.81, 95% CI 1.14 to 2.88, maternal age ≥40 (aOR 2.88, 95% CI 1.31 to 6.32), BMI (aOR 1.04, 95% CI 1.01 to 1.08, that is a 4% increase in risk for every unit increase in BMI), and SGA (aOR 2.76, 95% CI 1.59 to 4.80) (Table 3). The area under the ROC curve for the model was 0.736. Using non-left versus left in the multivariable model resulted in non-significant increase in late stillbirth risk compared with the combined non-left positions (aOR 1.35, 95% CI 0.92 to 1.99).

Among participants who reported going-to-sleep supine on the last night, 70.7% reported that their usual going-to-sleep position in the last week was also supine. Accordingly women with late stillbirth were also more likely to go to sleep supine in the last week (aOR 3.46, 95% CI 1.49 to 8.03) but risk was not increased with the combined non-left going-to-sleep positions in the last week (aOR1.04, 95% CI 0.74 to 1.48) (Table 3).

The population attributable risk for potentially modifiable risk factors associated with late stillbirth in our model were calculated (Table 4), with PAR for supine going-to-sleep position on the last night (9.4%) similar to risk with maternal overweight (8.4%).

**Table 4:** Late gestation stillbirth risk attributable to potentially modifiable risk factors in this study population.

Potentially modifiable risk factor	Prevalence in controls	Attributable risk
SGA	12.8%	24.7%
Maternal obesity (BMI ≥ 30)	23.7%	20.4%
Maternal overweight (BMI 25–29.9)	25.3%	8.4%
Supine going-to-sleep position last night	3.9%	9.4%
Maternal age ≥ 40 years	3.5%	5.4%

SGA: small for gestational age = birthweight <10^th^ customised centile

We further explored the risk associated with supine going-to-sleep position on the last night and during the last week in term (≥37 weeks’ gestation) and preterm (≥28 to 36 weeks’ gestation) cases and controls. The risk was greater for term (aOR 10.26, 3.00–35.04) than preterm stillbirths (aOR 3.12, 0.97–10.05) for the last night and also the last week (aOR 12.73 (2.92 to 55.46) vs aOR 2.25 (0.65 to 7.84) (Table 5).

**Table 5:** Analysis of going-to-sleep position and late stillbirth stratified by preterm and term gestation.

Preterm (≥28 to 36 weeks’) gestation						
Characteristic	Cases (n = 68)			Controls (n = 252)	Unadjusted odds ratio (95% CI)	Adjusted odds ratio (95% CI)
Going-to-sleep position on the last night *						
Left side	34 (50.0)			147 (58.3)	1.00	1.00
Non-Left	33 (48.5)			104 (41.3)	1.37 (0.80 to 2.36)	
Right side	22 (32.4)			86 (34.1)	1.11 (0.61 to 2.01)	0.96 (0.48 to 1.94)
Restless	4 (5.9)			4 (1.6)	4.32 (1.03 to 18.16)	3.50 (0.61 to 19.97)
Supine	6 (8.8)			13 (5.2)	2.00 (0.71 to 5.63)	3.12 (0.97 to 10.05)
Propped	1 (1.5)			1 (0.4)	4.32 (0.26 to 70.87)	4.37 (0.11 to 178.86)
Going-to-sleep position on the last week ^#^						
Left side	38 (55.9)			137 (54.4)	1.00	1.00
Non-Left	30 (44.1)			114 (45.2)	0.95 (0.55 to 1.63)	
Right side	19 (27.9)			75 (29.8)	0.91 (0.49 to 1.70)	0.73 (0.34 to 1.54)
Variable side	4 (5.9)			27 (10.7)	0.53 (0.18 to 1.62)	0.63 (0.18 to 2.19)
Supine	5 (7.4)			11 (4.4)	1.64 (0.54 to 5.01)	2.25 (0.65 to 7.84)
Propped	1 (1.5)			1 (0.4)	3.61 (0.22 to 59.00)	4.01 (0.08 to 210.43)
On front	1 (1.5)			0	NA	NA
Term (≥37 weeks’) gestation						
Characteristic	Cases (n = 96)			Controls (n = 317)	Unadjusted odds ratio (95% CI)	Adjusted odds ratio (95% CI)
Going-to-sleep position on the last night ^†^						
Left side	44 (45.8)			181 (57.1)	1.00	1.00
Non-Left	48 (50.0)			136 (42.9)	1.45 (0.91 to 2.31)	
Right side	22 (22.9)			101 (31.9)	0.90 (0.51 to 1.58)	0.98 (0.48 to 1.99)
Restless	10 (10.4)			18 (5.7)	2.29 (0.99 to 5.30)	2.00 (0.64 to 6.21)
Supine	13 (13.5)			9 (2.8)	5.94 (2.39 to 14.8)	10.26 (3.01 to 35.04)
Propped	3 (3.1)			8 (2.5)	1.54 (0.39 to 6.05)	1.02 (0.17 to 5.97)
Going-to-sleep position in the last week ^‡^						
Left side	47 (49.0)			165 (52.1)	1.00	1.00
Non-Left	48 (50.0)			152 (47.9)	1.11 (0.70 to 1.75)	
Right side	23 (24.0)			96 (30.3)	0.84 (0.48 to 1.47)	0.95 (0.48 to 1.89)
Variable side	11 (11.5)			45 (14.2)	0.86 (0.41 to 1.79)	1.11 (0.49 to 3.01)
Supine	10 (10.4)			5 (1.6)	7.02 (2.29 to 21.55)	12.73 (2.92 to 55.46)
Propped	4 (4.2)			6 (1.9)	2.34 (0.63 to 8.64)	2.64 (0.47 to 14.81)

Data are presented as number (percentage) or median (IQR). Two multivariable models stratified by preterm or term pregnancy have been conducted using either last night going-to sleep position or last week going-to-sleep position. Multivariable models are adjusted for gestation at interview in controls and at diagnosis of stillbirth for cases, district health board, and all the other variables in the Table 3.

* One preterm case and one preterm had unknown sleep position on the last night and were excluded from the multivariable model

^#^ One preterm control did not recall her sleep position in the last week and was excluded from the multivariable model

^†^ Four term cases had unknown sleep position on the last night and were excluded from the multivariable model

^‡^ One term case did not recall her sleep position in the last week and was excluded from the multivariable model

## Discussion

In this New Zealand multicentre case-control study, women in the third trimester of pregnancy who went to sleep supine the night before the baby was thought to have died, had an overall 3.7 fold increased risk of late stillbirth with a population attributable risk of 9.4%. This risk was independent of other common risk factors for late stillbirth such as obesity, smoking, advanced maternal age, and SGA. The consistent findings from two New Zealand case-control studies [8] with very similar methodology, an Australian and Ghanaian study [21, 22], as well as the biological plausability, suggest that the relationship between supine going-to-sleep position and late stillbirth is likely to be causal with applicability in both Western and non-Western settings. In addition a novel feature of our study is the finding that the risk associated with supine-going-to-sleep position is higher in term pregnancies compared with those between 28 and 36 weeks’ which may have implications for public health messages.

### Biological plausibility of findings

Reduced vena-caval diameter has been demonstrated by magnetic resonance imaging in supine compared with the left lateral position in late pregnancy [23]. Another study, using Doppler ultrasound, showed that blood flow in the uterine artery was less in the supine position than in the left lateral [10]. Adverse fetal effects of the supine position are suggested by reduced middle cerebral artery Doppler resistance, a fetal response to hypoxia [11], and reduced fetal oxygen saturation in labour in the supine position [24]. Furthermore we have recently reported that in healthy late pregnancy, when the mother is in the supine position, the fetus spends more time in behavioural state 1 (fetal quiescence) and less time in active fetal behavioral state 4, compared to when the mother is on her left side [25]. This provides additional evidence to support a relatively hypoxic environment for the fetus when a healthy mother is in the supine position in late pregnancy.

Supine position is also associated with sleep disturbed breathing, which increases in pregnancy, and has been associated with pregnancy complications including hypertensive disorders, gestational diabetes and fetal growth restriction [26]. Hence there are multiple mechanisms which could mediate the effects of supine position in late pregnancy on fetal wellbeing.

### Comparison with other studies

Compared with controls in the Auckland Stillbirth Study [8], there has been an increase in left sided going-to-sleep position (43% versus 57.6%) and a small decrease in supine going-to-sleep position (5% versus 3.8%) in the current study, which demonstrates that going-to-sleep position is modifiable. In addition we have recently completed a survey of sleep practices in late pregnancy in a multi-ethnic community with high perinatal mortality [27]. A large majority of women surveyed (227/263, 86%) who reported they did not currently go-to-sleep on their left side responded that they could change to going-to-sleep on their left side if this was better for their baby [27].

In the current study, the risk of late stillbirth was not elevated in women who reported going-to-sleep on their right on the last night and the last week; whereas in our previous study there was a borderline increase in risk with right sided compared with left sided going-to-sleep position on the last night [8]. Further evidence is required to confirm whether right sided going-to-sleep position is associated with similar risk of late stillbirth to left sided going-to-sleep position.

Contrary to our hypothesis, we did not demonstrate an association between sleeping during the day or long night-time sleep duration and late stillbirth risk. We did demonstrate an association between short sleep duration on the last night and late stillbirth risk (aOR 1.81, 95% CI 1.14 to 2.88), similar to findings in The Auckland Stillbirth Study. One possible explanation for this association is that women with short sleep duration may have been experiencing discomfort from symptoms of impending labour on the last night.

Consistent with data from meta-analyses of stillbirth risk factors [6, 7], the highest population attributable risks for late stillbirth were associated with SGA infants (24.7%) and maternal obesity (20.4%). The large majority of SGA stillborn infants are not currently recognised as SGA before birth [28]. Programmes that hold promise for reducing mortality in SGA pregnancies, such as the UK based Growth Assessment Protocol [29], require further evaluation. Consistent with a recent study [30], we demonstrated a dose dependent association between increasing BMI and stillbirth risk. Multiple mechanisms are likely responsible for the association between obesity and stillbirth [31]. Our findings again highlight the importance of entering pregnancy with a normal BMI to optimise the likelihood of a healthy pregnancy.

### Strengths and limitations of study

A case-control study is the most appropriate study design to address our hypothesis. As late stillbirth is relatively rare (3/1000 births), a prospective cohort study design is not feasible or affordable. Such a design would involve recruitment of hundreds of thousands of women with regular completion of questionnaires about sleep practices and other lifestyle factors.

In any case-control study there is potential for recall bias. This was reduced by using a structured interview and ensuring that participants and research midwives were not informed of specific study hypotheses. Information about going-to-sleep position was by necessity collected by self-report. The reliability of self-reported sleep position has been questioned [32], however, our group has recently demonstrated that there is good correlation between maternal short term recall of going-to-sleep position and going-to-sleep position recorded by video technology [33].

The median time to interview in women with late stillbirth was 24 days. This is unlikely to have resulted in recall bias which is a systematic difference between cases and controls in the accuracy of recalled information. The time lapse could influence the accuracy of recalled information but this would not be biased towards the exposure.

Maternal report was also used to estimate the timing of fetal death, with potential that in some cases the ‘last night’ was not the night before fetal death, or the night during which the baby died. However, a similar risk of late stillbirth was seen for women who reported they usually went to sleep supine in the last week (aOR 3.46, 95% CI 1.49 to 8.03) as for the last night. Furthermore 70.7% of women who reported going-to-sleep supine on the last night also reported usually going-to-sleep supine in the last week. This suggests that self-report of ‘last night’ is likely to be reliable and adopting a supine going-to-sleep position on the last night is not a response to the baby’s death.

During recruitment there was a reduction in late stillbirths after 37 weeks of gestation in New Zealand resulting in fewer cases recruited compared with projected numbers. With this decrease in late stillbirth we were underpowered to investigate interactions between going-to-sleep position and measures of fetal vulnerability such as small for gestational age infants and maternal obesity and smoke exposure [34]. We used very similar study design and questionnaires as The Auckland Stillbirth Study [8] and the Midlands and North of England Stillbirth Study [35] and are therefore planning an individual participant data meta-analysis to investigate these interactions and also to determine whether right sided going-to-sleep position is associated with increased risk (PROSPERO registration: CRD42017047703).

Controls in our study were selected from the pregnant population at similar gestation to the stillbirths, allowing comparison of maternal lifestyle at similar gestational age. The participants were also broadly representative of the eligible population and our results are likely to be generalisable to other similar populations.

## Conclusions

Supine going-to-sleep position is a modifiable risk factor for late stillbirth. Reductions in non-left and supine going-to-sleep position over time in New Zealand suggest that women are able to modify their going-to-sleep position in response to advice. Although there has been no formal public health campaign in New Zealand, it is likely that midwives, obstetricians and childbirth educators are advising pregnant women to go to sleep on their left side. Public health education should be developed recommending that women in the third trimester do not settle to sleep supine, especially after 37 weeks’.

In conclusion, women in the third trimester of pregnancy who went to sleep supine the night before their baby died, had a 3.7 fold overall increased risk of late stillbirth that was independent of other common risk factors. These findings confirm those in our original hypothesis generating study and are biologically plausible, suggesting that the relationship may be causal. Successful public health interventions advising that women do not go to sleep supine in late pregnancy have potential to reduce late stillbirth by approximately 9% overall.

## Supporting information

S1 Appendix Questionaire for the New Zealand multicentre stillbirth case-control study. (DOC)
