# Supplementary material for: Going to sleep in the supine position is a modifiable risk factor for late pregnancy stillbirth; Findings from the New Zealand multicentre stillbirth case-control study
Source: PLoS One. 2017 Jun 13;12(6):e0179396. doi: 10.1371/journal.pone.0179396 (PMC5469491; doi:10.1371/journal.pone.0179396)
Supplement: S1 Appendix — (DOC) [file pone.0179396.s001.doc]

| Study Number | Multi-Centre Stillbirth Study | Date |
| --- | --- | --- |
| __ __ __ __ __ __ | **Maternal Interview** | ____/____/____ |

**Case**
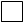
 **Control**
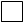
 ***Attached NHI?*** Yes
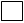
 No
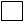


*(To be recorded at interview)* **Height in cms:** _________ **Weight in kgs:** ________

**DHB: ______________________ Midwife Interviewer: _____________________________________**

**Who else is present at interview?**  ___________________________________________________________

1. **Inclusion criteria**

**Cases:** Gestation greater than or equal to 28 weeks at time of stillbirth? *(Not at time of birth)*

Yes
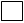
 No
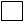


Stillbirth occurred around 1 to 5 weeks before interview? *(Not eligible if more than 6 weeks previously)*

Yes
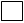
 No
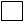
*(Reason)__________________________________*

**Controls:** Gestation **within 2 weeks of the gestation specified** at time of interview? *(Not eligible if given birth)*

Yes
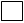
 No
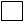


____________________________________________________________________________________________________________________________________________________________________________________________________________________________________________________________________________________________________________________________________________________________________________________________________________________________________________________________________

**Please Complete This Section for All Consented and Declined Cases and Controls**

**A.1**: **Gestation?** _______/_______ (weeks/days at time of interview/decline for controls and time of birth for cases)

***(NB: Please ensure this gestation agrees with Question 1.3, “Best Agreed EDD”)***

**A.2**: **Singleton pregnancy?**  Yes
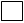
 No
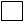


**A.3: Major fetal abnormality?** Yes
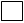
 No
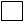


**A.4**: **Consent signed?** Yes
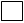
 No*
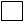
 *** If Not consented please specify reason*:***

Unable to contact?
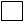


LMC declined?
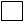


Woman declined?
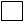


Woman missed interview?
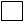


Other
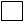
____________________________

______________________________________

****If NO consent obtained please complete basic clinical data*** *(all questions to/including B.3 on next page)* ***and record the woman’s EDD, Gravida/Parity and Initial LMC Type*** *(as per Q 1.8)* ***below:***

*EDD________________ G______ P_______ Initial LMC Type _______________________*

**A.5**: **Fluent in English?** Yes
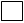
 No
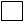


**A.5.1: If not fluent in English, was an interpreter used?** Yes
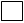
 No
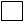
 NA
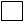


**B. Maternal demographics**

**B.1**: **What is your** d**ate of birth?**  ______/______/_______ (DD/MM/YYYY) Age _________ (years)

**B.2**: **Which country were you born in**? _______________________________________________

**B.2.1**: **If not New Zealand: how many years have you lived in New Zealand**? _________ (years)

**B.3: How do you describe your ethnicity? *(Please choose up to 3)***

| 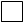Maori | 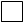New Zealand European |
| --- | --- |
| 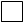Chinese | 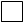Cook Islander |
| 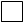Fijian | 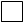Indian |
| 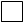Niuean | 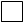Samoan |
| 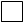Tongan | 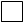Other Asian ____________________________ |
| 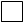Other European _____________________ | 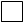Other Pacific Island ______________________ |
| 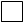Other ______________________________ |  |

**Interview form now completed for those declined or unable to be contacted**

**B.4:** **Usual residential address at time of interview *(for cases)* or birth *(for controls)***

*(This data is to enable decile coding only and will not be used to identify the woman)*

Address 1:_________ **(house number)**

Address 2: ________________________________________________________ **(street)**

Address 3: ________________________________________________________ **(suburb)**

Address 4: ________________________________________________________ **(town/city)**

**Postcode: ____________**

**B.5: What best describes the place that you* live in?** *(*lived in most of the time you were pregnant)*

1. Own house
2. Private rental
3. Government or state house or council rental
4. Stay with family or friends

99 Other ____________________________________________________________________

**B.6: How many people usually* live in your house?** *(*lived in your house while you were pregnant)*?

1. Children under 10 year’s _______ (number of children)

2. Couples (including you) _______ (number of couples)

3. Other adults and children over 10 year’s _______ (number of other adults and children)

B.7: How many bedrooms* does your house have? _______ (number of bedrooms)

(*did the house have that you lived in while you were pregnant have)?

B.8: Do* you feel your house is large enough for your family’s needs?

(*Did you feel the house you lived in while you were pregnant was large enough for your family’s needs?)

Yes
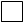
 No
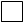


**B.9: What is your highest educational level?**

***(Please circle one answer only)***

1. Completed primary school
2. Completed years 8 – 10 or Form 2 – 4
3. Completed year 11 or Form 5 (NCEA 1/School Certificate)
4. Completed year 12 or Form 6 (NCEA 2/University Entrance)
5. Completed year 13 or Form 7 (NCEA 3/School Bursary)
6. Trade certificate or similar
7. University or Tertiary institute or Polytechnic degree
8. Other (please specify)_______________________________________________________________

**B.10: What was your work situation prior to this pregnancy?** *(Please circle one answer only)*

1. Full time work (over 20 hours per week)
2. Part time work (includes casual work)
3. Student
4. Home maker
5. Unemployed
6. Sickness beneficiary
7. Other (please specify)_______________________________________________________________

**B.11: What was your work situation in the last month*?** *(Please circle one answer only)*

*(*the last month before your baby died)*

1. Full time work (over 20 hours per week)
2. Part time work (includes casual work)
3. Student
4. Home maker
5. Unemployed
6. Sickness beneficiary
7. Maternity leave

99 Other (please specify) ______________________________________________________________

**B.12: What was your partner's** (not necessarily father of the baby) **work situation in the last month*?**

*(*the last month before your baby died)*

- - 1. Full time work (over 20 hours per week)
    2. Part time work (includes casual work)
    3. Student
    4. Home maker
    5. Unemployed
    6. Sickness beneficiary

1. Other/unknown/no partner (please specify) ___________________________________________

**C. Relationships**

C.1: What is your marital status? (Please circle one answer only)

1. Single (does not live with a partner but may live with family/friends)
2. Married
3. Defacto

C.2: How old is the father of this baby? ________ (years) Don't know
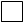


C.3: How would he describe his ethnicity? (Please choose up to 3 answers)


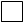
Unknown


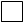
 Maori


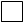
 New Zealand European


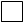
 Chinese


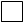
 Cook Islander


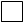
 Fijian


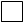
 Indian


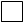
Niuean


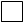
 Samoan


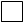
 Tongan


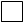
 Other Asian (please specify) _______________________________________________________


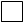
 Other European (please specify) ____________________________________________________


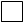
 Other Pacific Island (please specify) _________________________________________________


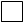
 Other (please specify) ____________________________________________________________

C.4: Is this your first pregnancy with the father of this baby? Yes
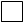
 No
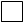


C.5: How long had you had a relationship with the father of this baby when you conceived?

(Please circle one answer only)

1. Conceived on first episode of intercourse
2. Less than 6 months
3. 6-12 months
4. More than 1 year
5. Declined to answer

99 Other (specify) _____________________________________________________________________

**D. General Health and Past History**

**D.1: Do you have any medical conditions?** *(Please tick all relevant answers)*


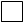
None


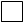
Anaemia (prior/booking Hb <100g/L)
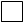
Systemic lupus erythematosus


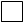
Asthma
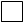
Thalassaemia


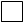
Cervical surgery
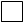
Thrombophilia


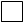
Depression
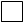
UTI recurrent


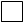
Diabetes NIDDM
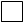
Uterine abnormality


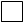
Diabetes IDDM
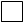
Uterine surgery


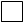
Epilepsy
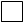
Venous thromboembolism


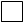
Heart condition congenital
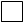
Other medical condition (specify) ________________


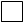
Heart condition rheumatic ___________________________________________


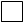
Hypertension (Essential or Chronic)


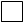
Hyperthyroid


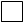
Hypothyroid


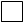
Inflammatory bowel disease (Crohn’s disease or ulcerative colitis)


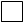
Polycystic ovarian syndrome


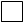
Psychiatric disorder (other than depression)


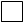
Renal disease

D.2: Did you have fertility treatment to become pregnant with this baby? Yes
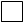
 No
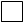


D.2.1: If Yes, what was the treatment? (Please circle one answer only)

- 1. Artificial insemination
  2. Ovulation induction
  3. IVF
  4. GIFT
  5. ICSI intracytoplasmic sperm injection

99 Other (specify) __________________________________________________________________

***Gravida**** *______* ***Para**** *______ (*Prior to stillbirth)*

**D.3 Have you ever been pregnant before?** Yes
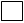
 No
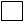
 **(Please go directly to next page to Q D.4)**

**If Yes:**

**D.3.1 How many were miscarriages or ectopic pregnancies in the first 12 weeks of pregnancy?**

Nil
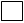
 _________ (number)

**D.3.2 How many were miscarriages or ectopic pregnancies between 13 and 20 weeks of pregnancy?**

Nil
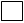
 _________ (number)

**D.3.3 Have you had any terminations of pregnancy under 20 weeks?**

Nil
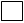
 _________ (number)

**D.3.4 Have you had any other pregnancies:** Yes
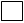
 No
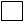
 (**If No, go to next page to Q D.4)**

**If Yes, can you tell me about your other pregnancies and births*? *(*Do not include current pregnancy/stillbirth)***

| Year of birth | Gestation | Birth weight  (in gms) | Outcome |  | | | | |
| --- | --- | --- | --- | --- | --- | --- | --- | --- |
|  |  |  | LB/SB/NND |  |  |  |  |  |
|  |  |  | LB/SB/NND |  |  |  |  |  |
|  |  |  | LB/SB/NND |  |  |  |  |  |
|  |  |  | LB/SB/NND |  |  |  |  |  |
|  |  |  | LB/SB/NND |  |  |  |  |  |
|  |  |  | LB/SB/NND |  |  |  |  |  |
|  |  |  | LB/SB/NND |  |  |  |  |  |
|  |  |  | LB/SB/NND |  |  |  |  |  |
|  |  |  | LB/SB/NND |  |  |  |  |  |

**List other births:**____________________________________________________________________________

###### **D.3.4.1 Did you have a caesarean section for any of these births?** Yes
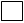
 No
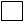


**Antenatal Care**

**D.4:** **How many weeks pregnant were you when you first saw a health professional about this pregnancy?**

**_________** (weeks)

**D.5: Who did you first se**e? (*Please circle one answer only)*

1. GP
2. Midwife
3. Infertility specialist
4. Private obstetrician
5. Hospital obstetrician
6. Family planning clinic
7. Pharmacist
8. Nurse
9. Other: ­­­­­­­­­­­­­­­­­­­­­­_________________________________________________________________________

**D.6: How many weeks pregnant were you when you first saw your Lead Maternity Carer (the person who is**

**responsible for your maternity care)?**

**_________** (weeks)

**D.7: If your GP is not your Lead Maternity Carer did you also see a GP (including at an emergency after**

**hours centre)** **during your pregnancy?**

(Exclude pregnancy visits to GP prior to obtaining LMC unless this was a formal “Shared Care” arrangement)

Yes
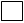
 No
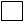
 **(If No, go to Q D.8)**

**D.7.1 If Yes, how many times did you see a GP?** **_________** (number of GP visits)

**D.7.2 Were any of these GP visits for routine antenatal care?**

(Exclude pregnancy visits to GP prior to obtaining LMC unless this was a formal “Shared Care”

arrangement)

**_________** (number of GP visits)

**D.8: Did you have a flu vaccination in pregnancy?** Yes
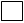
 No
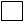


**D.9: Did you have any of these common illnesses/problems during your pregnancy*?**

|  | **Anytime during your pregnancy** | **Last two weeks of your pregnancy***  ***(*before your baby died)*** |
| --- | --- | --- |
| **High fever** | Yes 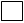 No 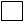 | Yes No |
| **If Yes, was the high fever confirmed to be higher than 38c by thermometer** | Yes No | Yes No |
| **Runny nose/sore throat/swollen glands** | Yes No | Yes No |
| **Cough with phlegm** | Yes No | Yes No |
| **Diarrhoea and/or vomiting** | Yes No | Yes No |
| **Frequent urge to urinate and/or pain on urinating** (UTI symptoms) | Yes No | Yes No |

**D.10: *Have you been unwell in any other way in the last two weeks?**

*(*Were you unwell in any other way in the last two weeks of your pregnancy before your baby died?)*

Yes No

**D.10.1: If yes, please describe:** __________________________________________________________

_____________________________________________________________________________________

_____________________________________________________________________________________

_____________________________________________________________________________________

_____________________________________________________________________________________

_____________________________________________________________________________________

**D.11: Did you have any vaginal bleeding in your pregnancy?**

*(Please circle one answer only)*

01 No bleeding

02 Single episode < 20 weeks

03 Recurrent bleeds < 20 weeks

04 Single >20 weeks

05 Recurrent bleeds >20 weeks

06 Recurrent bleeds throughout

99 Unsure

**D.12**: **During your pregnancy, did you take any antibiotics**?

*(Please refer to Appendix VII: Antibiotics Commonly Prescribed in NZ General Practice if woman is unsure)*

Yes No

**D.12.1: If Yes, what antibiotic and what did you have it for?** _____________________________________________________________________________________

_____________________________________________________________________________________

_____________________________________________________________________________________

_____________________________________________________________________________________

_____________________________________________________________________________________

**D.12.2: How many weeks pregnant were you when you took the antibiotic?** ________ (weeks)

*If more than one course of antibiotics in pregnancy, record the gestation at the time of the most recent)*

**E. Diet**

**E.1: In the first three months of pregnancy how often did you eat a serving of the following foods?**

***(Please circle one answer for each food)***

|  | **Not at all** | **Less than once a week** | **Once a week** | **A few times a week** | **Once a day** | **A few times a day** |
| --- | --- | --- | --- | --- | --- | --- |
| Apples/pears | 1 | 2 | 3 | 4 | 5 | 6 |
| Kiwifruit | 1 | 2 | 3 | 4 | 5 | 6 |
| Citrus fruit | 1 | 2 | 3 | 4 | 5 | 6 |
| Bananas | 1 | 2 | 3 | 4 | 5 | 6 |
| Dried fruit | 1 | 2 | 3 | 4 | 5 | 6 |
| Green vegetables | 1 | 2 | 3 | 4 | 5 | 6 |
| Root vegetables | 1 | 2 | 3 | 4 | 5 | 6 |
| Peas/maize/lentils | 1 | 2 | 3 | 4 | 5 | 6 |
| Fried rice/noodles | 1 | 2 | 3 | 4 | 5 | 6 |
| Boiled rice/pasta | 1 | 2 | 3 | 4 | 5 | 6 |
| Fish/shellfish | 1 | 2 | 3 | 4 | 5 | 6 |
| Soup with meat | 1 | 2 | 3 | 4 | 5 | 6 |
| Jelly | 1 | 2 | 3 | 4 | 5 | 6 |
| Ice cream | 1 | 2 | 3 | 4 | 5 | 6 |
| Sweet biscuits | 1 | 2 | 3 | 4 | 5 | 6 |
| Cakes | 1 | 2 | 3 | 4 | 5 | 6 |
| Crisps | 1 | 2 | 3 | 4 | 5 | 6 |
| Pies | 1 | 2 | 3 | 4 | 5 | 6 |
| Chocolate bars | 1 | 2 | 3 | 4 | 5 | 6 |
| Lollies | 1 | 2 | 3 | 4 | 5 | 6 |
| Ice blocks | 1 | 2 | 3 | 4 | 5 | 6 |
| Milk | 1 | 2 | 3 | 4 | 5 | 6 |

**E.2: During the last four weeks (of pregnancy)* how often did you eat a serving of the following foods?**

***(Please circle one answer for each food)* (*before)**

|  | **Not at all** | **Less than once a week** | **Once a week** | **A few times a week** | **Once a day** | **A few times a day** |
| --- | --- | --- | --- | --- | --- | --- |
| Apples/pears | 1 | 2 | 3 | 4 | 5 | 6 |
| Kiwifruit | 1 | 2 | 3 | 4 | 5 | 6 |
| Citrus fruit | 1 | 2 | 3 | 4 | 5 | 6 |
| Bananas | 1 | 2 | 3 | 4 | 5 | 6 |
| Dried fruit | 1 | 2 | 3 | 4 | 5 | 6 |
| Green vegetables | 1 | 2 | 3 | 4 | 5 | 6 |
| Root vegetables | 1 | 2 | 3 | 4 | 5 | 6 |
| Peas/maize/lentils | 1 | 2 | 3 | 4 | 5 | 6 |
| Fried rice/noodles | 1 | 2 | 3 | 4 | 5 | 6 |
| Boiled rice/pasta | 1 | 2 | 3 | 4 | 5 | 6 |
| Fish/shellfish | 1 | 2 | 3 | 4 | 5 | 6 |
| Soup with meat | 1 | 2 | 3 | 4 | 5 | 6 |
| Jelly | 1 | 2 | 3 | 4 | 5 | 6 |
| Ice cream | 1 | 2 | 3 | 4 | 5 | 6 |
| Sweet biscuits | 1 | 2 | 3 | 4 | 5 | 6 |
| Cakes | 1 | 2 | 3 | 4 | 5 | 6 |
| Crisps | 1 | 2 | 3 | 4 | 5 | 6 |
| Pies | 1 | 2 | 3 | 4 | 5 | 6 |
| Chocolate bars | 1 | 2 | 3 | 4 | 5 | 6 |
| Lollies | 1 | 2 | 3 | 4 | 5 | 6 |
| Ice blocks | 1 | 2 | 3 | 4 | 5 | 6 |
| Milk | 1 | 2 | 3 | 4 | 5 | 6 |

**E.3: Did you have “morning sickness” this pregnancy?** Yes No

**E.3.1: If Yes, were you admitted to hospital due to your vomiting?** Yes No

**E.4: Did you take supplements this pregnancy?**

|  | **Months prior to pregnancy** | **First 3 months of pregnancy** | **In the last month of pregnancy***  *(*before your baby died)* |
| --- | --- | --- | --- |
| **Multivitamins**  (contain multiple vitamins and dietary minerals) | Yes No  If Yes, name/amount daily: | Yes No  If Yes, name/amount daily: | Yes No  If Yes, name/amount daily: |
| **Folic Acid*** | Yes No  If Yes, name/amount daily:  **Total:** | Yes No  If Yes, name/amount daily:  **Total:** | Yes No  If Yes, name/amount daily:  **Total:** |
| **Iodine** | Yes No  If Yes, name/amount daily: | Yes No  If Yes, name/amount daily: | Yes No  If Yes, name/amount daily: |

**Please see Appendix VIII: Folic Acid Content of Commonly Used Vitamin/Mineral Preparations to work out total folic acid intake which may be from a combination of multivitamins/iron-folic acid medications*

**F. Personal habits**

**F.1: Do you currently smoke cigarettes?** *(Please circle one answer only)*

01 Yes

02 No, stopped in this pregnancy

03 No, stopped prior to this pregnancy **(If No, please go directly to Q F.5)**

04 No, never smoked **(If No, please go directly to Q F.5)**

**F.1.2: If you smoked at any time in this pregnancy, how many cigarettes a day on average did you**

**smoke?** ________ (cigarettes)

**F.2:** **If you stopped smoking in this pregnancy: how many weeks pregnant were you when you stopped**?

*________* (weeks)

**F.3: Were you referred to Smokechange (or equivalent)** Yes No

(Tick ‘yes’ if referred - even if did not take part)

**F.4: Did you use nicotine patches, gum or lozenges in pregnancy?** Yes No

F.5: Does your partner smoke? No partner Yes No

F.6: Does anyone else who lives in your house smoke? Yes No

F.7: On average, how many standard alcoholic drinks (if any) do* you have each week?

(*did you have each week before your baby died)

|  | **First 3 months of pregnancy** | **In the last month of pregnancy** |
| --- | --- | --- |
| **Nil drinks** |  |  |
| **< 1 std drink wk** |  |  |
| **1-2 std drinks wk** |  |  |
| **3-4 std drinks wk** |  |  |
| **≥5 std drinks wk** |  |  |

Please see Appendix IX to work out standard drinks per week

F.8: What were the most standard alcoholic drinks that you had on any one occasion during your

pregnancy*? (*before your baby died)

(Please circle one answer only)

01 None

02 1 to 2

03 3 to 4

04 5 to 10

05 Greater than 10

F.8.1: How many weeks pregnant were you when you had the most drinks? ________ (weeks)

F.9: At any stage in your pregnancy did you use any of the following drugs*? (*before your baby died)

No (If No, please go directly to Sleep Practices on next page)

Yes

If Yes, please indicate the type of drug used and when it was used

|  | **First 3 months of pregnancy** | **In the last month of pregnancy** | **In the last week of pregnancy** |
| --- | --- | --- | --- |
| **Herbal highs** |  |  |  |
| **Cannabis** |  |  |  |
| **Amphetamine/P** |  |  |  |
| **Ecstasy** |  |  |  |
| **Cocaine** |  |  |  |
| **Heroin** |  |  |  |
| **Methadone** |  |  |  |
| **Other drugs** |  |  |  |

F.9.1: If Yes, how often did you use these drugs? (Please circle one answer only)

1. Daily use
2. Weekly use
3. Occasional use
4. Once only

99 Other (specify) _________________________________________________________________

G. Sleep Practices

**Sleep duration and latency**

**G.1: On average how long do you think it usually took you to fall asleep each night in the last week*?**

*(*the last week before your baby died)*

_______ (minutes)

**G.1.1: Last night*, how long do you think it took you to fall asleep?** *(*the last night before your baby died)*

_______ (minutes)

**G.2: On average how many hours of actual sleep did you get at night in the last week*?**

*(*the last week before your baby died)*

_______ (hours) _______ (minutes)

**G.2.1: Last night*, how many hours of actual sleep did you get** *(*the last night before your baby* *died)*

_______ (hours) _______ (minutes)

**Insomnia**

**G.3: Did you have difficulty getting to sleep in the last week*?** *(*the last week before your baby died)*

(Please circle one answer only)

01 Never

02 Rarely

03 Sometimes

04 Often

05 Every night

**G.3.1: Last night* did you have difficulty getting to sleep?** *(*the last night before your baby died)*

Yes No

**G.4: Did you regularly wake up during the night or early morning in the last week*** *(*the last week before your baby died)*

Yes No

**G.4.1: If Yes, how many times?**  ________ (times)

**G.5: Last night* did you wake during the night or early morning?** *(*the last night before your baby died)*

Yes No

**G.5.1: If Yes, on average how many times?** ________ (times)

**G.6: If you woke up in the night did you get up go to the toilet or for any other reason** **in the last week*?**

*(*the last week before your baby died)*

Yes No

**G.6.1: If Yes, on average how many times?** _______ (times)

**G.7: If you woke up in the night did you get up go to the toilet or for any other reason** **last night*?**

*(*the last night before your baby died)*

Yes No

**G.7.1: If Yes, how many times?**  _______ (times)

**G.8: In the last week* did you have difficulty getting back to sleep if you woke in the night**

*(*the last week before your baby died)*

*(Please circle one answer only)*

1. Never
2. Rarely
3. Sometimes
4. Often
5. Every night

**G.8.1: Last night*, if you woke did you have difficulty getting back to sleep?**

*(*the last night before your baby died)*

Yes No Didn’t wake

# Bed

**G.9: What size bed did you sleep in last night*?**

(**the last night before your baby died?)*

*(Please circle one answer only)*

1. King
2. Queen
3. Double
4. King single
5. Standard single
6. Other-didn’t sleep in bed (specify)_______________________________________________________

**G.10: Did anyone else sleep in the same bed as you last night*?**

*(*the last night before your baby died)*

Yes partner Yes other ________________ No

**G.11: Which side of the bed did you sleep on last night*?**

*(*the last night before your baby died)*

Side of bed determined by woman when she is lying on her back in bed

*(Please circle one answer only)*

1. Left
2. Middle
3. Right
4. Unsure

**G.12**: **How many pillows did you usually use at night in the last week*?**

*(*the last week before your baby died)*

**_________** *(number of pillows)*

**G.13: Did you sleep propped up in the last week*?**

*(*the last week before your baby died)*

Yes No  Unsure

**G.13.1: Did you sleep propped up last night?**

*(*the last night before your baby died)*

Yes No

**Sleep position**

**G.14: What position did you usually fall asleep in?**

*(Please circle one answer per line)*

|  | Left side | Back | Right side | Tummy | Variable | Propped  up | Don’t remember |
| --- | --- | --- | --- | --- | --- | --- | --- |
| Last week* | 1 | 2 | 3 | 4 | 5 | 6 | 99 |
| Last night* | 1 | 2 | 3 | 4 | 5 | 6 | 99 |

*(*the last week before your baby died* **or** **the last night before your baby died)*

**G.15: What position did you usually wake up in?**

*(Please circle one answer per line)*

|  | Left side | Back | Right side | Tummy | Variable | Propped  up | Don’t remember |
| --- | --- | --- | --- | --- | --- | --- | --- |
| Last week* | 1 | 2 | 3 | 4 | 5 | 6 | 99 |
| Last night***** | 1 | 2 | 3 | 4 | 5 | 6 | 99 |

*(*the last week before your baby died* **or** **the last night before your baby died)*

**G.16: Did you change sleep position during the night?**

*(Please circle one answer per line)*

|  | Not at all | Possibly once | Possibly twice | More than twice but not lots | Lots of times |
| --- | --- | --- | --- | --- | --- |
| Last week***** | 1 | 2 | 3 | 4 | 5 |
| Last night***** | 1 | 2 | 3 | 4 | 5 |

*(*the last week before your baby died* **or** **the last night before your baby died)*

**G.17: Would you describe yourself as a restless sleeper (i.e. move a lot during the night)?**

*(Please circle one answer per line)*

|  | Not at all | A little | Average | More than average | Very restless |
| --- | --- | --- | --- | --- | --- |
| Last week***** | 1 | 2 | 3 | 4 | 5 |
| Last night***** | 1 | 2 | 3 | 4 | 5 |

*(*the last week before your baby died* **or** **the last night before your baby died)*

**G.17.1 What sleep position did you usually** find the most comfortable?

|  | Left side | Back | Right side | Tummy | Variable | Propped  up | Don’t remember |
| --- | --- | --- | --- | --- | --- | --- | --- |
| Last week* | 1 | 2 | 3 | 4 | 5 | 6 | 99 |
| Last night***** | 1 | 2 | 3 | 4 | 5 | 6 | 99 |

*(*the last week before your baby died* **or** **the last night before your baby died)*

**G.17. 2 What, if any, advice have you been given by your doctor or midwife about what position you should sleep in during the last three months of pregnancy?** *(Please circle one answer only)*

01 No information about sleep position

02 Not to be concerned about my sleep position

03 Not to sleep on my back

04 To sleep on either side

05 To sleep on my left side

06 To sleep on my right side

99 Other (specify) _____________________________________________________________________

**G. 17.3 Other than information from your doctor or midwife, what have you read or heard about the best position to sleep in during late pregnancy?** *(Please circle one answer only)*

01 No information about sleep position

02 Not to be concerned about my sleep position

03 Not to sleep on my back

04 To sleep on either side

05 To sleep on my left side

06 To sleep on my right side

99 Other (specify) _____________________________________________________________________

**G. 17.3.1** Where did you read or hear this information? (*Please select all answers that apply)*

01 Radio/TV

02 Newspaper

03 Internet

04 Family and friends

05 Childbirth educator

06 Book or pamphlet

99 Other (specify) _______________________________________________________________

**Snoring**

**G.18: Have you EVER been told that you snore, or have woken yourself up snoring?**

Yes No (If No, please go directly to p.17, G.24. Restless Leg Syndrome)

**If Yes…**

**G.19: If you have been told you snore, or you have woken yourself up snoring, how often did this occur in**

**the last week*?** *(*the last week before your baby died) (Please circle one answer only)*

1. Never
2. Rarely
3. Sometimes
4. Often
5. Every night

**G.19.1: Did you snore last night?** (**the last night* *before your baby died)*

Yes No  Don’t know

**G.20: Did your snoring ever bother other people in the last week?** (**the last week* *before your baby died)*

Yes No  Don’t know

**G.20.1: Did your snoring bother anyone last night?** (**the last night* *before your baby died)*

Yes No  Don’t know

***G.21:*** ***How loud on average is your snoring reported to be?*** *(Please circle one answer per line)*

|  | Slightly louder than breathing | As loud as talking | Louder than talking | Very loud, can be heard in adjacent rooms | Don’t know |
| --- | --- | --- | --- | --- | --- |
| Last week***** | 2 | 3 | 4 | 5 | 99 |
| Last night***** | 2 | 3 | 4 | 5 | 99 |

*(*the last week before your baby died* **or** **the last night before your baby died)*

**G.22: Have you been told you briefly stop breathing when you are asleep in the last week*?**

*(*the last week before your baby died) (Please circle one answer only)*

1. Never
2. Rarely
3. Sometimes
4. Often
5. Every night

**G.22.1: Were you told you briefly stopped breathing when asleep last night*?**

(**the last night* *before baby died)*

Yes No

**G. 23: Have you been told you cough or choke during sleep in the last week*?**

*(*the last week of pregnancy before your baby died)* *(Please circle one answer only)*

1. Never
2. Rarely
3. Sometimes
4. Often
5. Every night

**G.23.1: Were you told you coughed or choked during sleep last night*?**

(**the last night* *before your baby died)*

Yes No

**Restless leg syndrome**

**In the last week*** *(*the last week before your baby died)* ***…..***

**G.24: Did you have unpleasant sensations (such as creepy, crawly or tingly feelings) in your legs**

**combined with an urge or need to move your legs?**

Yes No (**If No, please go directly to G.25 Sleepiness)**

**G.24.1: If Yes, did these feelings occur mainly or only at rest and do they improve with movement?**

Yes No

**G.24.2: Were these feelings (unpleasant sensation and need to move) worse in the evening or night**

**than in the morning?**

Yes No

**G.24.3: How often did these feelings (unpleasant sensation and need to move) occur in the last**

**week*?** *(*the last week before your baby died)* (Please circle one answer only)

01 Never

02 Occasionally

03 1-2 times a week

04 3-4 times a week

05 5-6 times a week

06 Every night

**Sleepiness**

**G.25: How would you rate your sleep quality overall?**

*(Please circle one answer per line)*

|  | Very good | Fairly good | Average | Fairly bad | Very bad |
| --- | --- | --- | --- | --- | --- |
| Last week***** | 1 | 2 | 3 | 4 | 5 |
| Last night***** | 1 | 2 | 3 | 4 | 5 |

*(*the last week before your baby died* **or** **the last night before your baby died)*

**G.26: How often do you feel tired or fatigued in the morning after your night’s sleep?**

*(Please circle one answer per line)*

|  | Never | Occasionally | 1-2 times per week | 3-4 times per week | 5-6 times per week | Every day | Don’t know |
| --- | --- | --- | --- | --- | --- | --- | --- |
| Last week* | 1 | 2 | 3 | 4 | 5 | 6 | 99 |

*(*the last week before your baby died)*

**G.26.1: Did you feel tired or fatigued after your sleep last night*?**

*(*the last night before your baby died)*

Yes No

**G.27: During your wake time in the day did you feel tired, fatigued or not up to par?**

*(Please circle one answer per line)*

|  | Never | Occasionally | 1-2 times per week | 3-4 times per week | 5-6 times per week | Every day | Don’t know |
| --- | --- | --- | --- | --- | --- | --- | --- |
| Last week * | 1 | 2 | 3 | 4 | 5 | 6 | 99 |

*(*the last week before your baby died)*

**G28: During the last week* how likely were you to doze off or fall asleep in the following**

**situations in contrast to just feeling tired?** *(*the last week before your baby died)*

Even if you have not done some of these things recently try and work out how they may have affected you.

*(Please circle one answer per line)*

| **During last week** | Would never doze | Slight chance of dozing | Moderate chance of dozing | High chance of dozing |
| --- | --- | --- | --- | --- |
| Sitting and reading | 1 | 2 | 3 | 4 |
| Watching TV | 1 | 2 | 3 | 4 |
| Sitting inactive in a public place (e.g. cinema, meeting, church) | 1 | 2 | 3 | 4 |
| **During last week** | Would never doze | Slight chance of dozing | Moderate chance of dozing | High chance of dozing |
| **Even if you have not done some of these things recently try and work out how they may have affected you** | | | | |
| As a passenger in a car for an hour without a break | 1 | 2 | 3 | 4 |
| Lying or sitting down to rest in the afternoon | 1 | 2 | 3 | 4 |
| Sitting and talking to someone | 1 | 2 | 3 | 4 |
| Sitting quietly after lunch without alcohol | 1 | 2 | 3 | 4 |
| In a car, while stopped for a few minutes in the traffic | 1 | 2 | 3 | 4 |

**G.29: On average how many times would you take a nap during the day?**

*(Please circle one answer per line)*

|  | Never | Occasionally | 1-2 times per week | 3-4 times per week | 5-6 times per week | Every day | Don’t know |
| --- | --- | --- | --- | --- | --- | --- | --- |
| Last week* | 1 | 2 | 3 | 4 | 5 | 6 | 99 |

*(*the last week before your baby died)*

**G.29.1: On average, how long would you nap for in the last week*?**

*(*the last week before your baby died)*

________ (hours) ________ (minutes)

**G.29.2: Did you take a nap during the day yesterday? *(Controls)***

*(Did you take a nap during the day before you thought your baby died?)* ***(Cases)***

Yes No

**G.30.2.1: If Yes, how long did you nap for?** _________ (hours) ________ (minutes)

**G.30: One hears about “morning” and “evening” types of people. Which one of these types do you**

**consider yourself to be?**

***(Please circle one answer only)***

| 1. Definitely a morning type 2. More a morning than an evening type 3. Neither more an evening than a morning type 4. Definitely an evening type |  |
| --- | --- |

###### **H. Fetal movements**

**H.1: Was there any time from 26 weeks of pregnancy that your baby’s movements were less than usual?**

Yes No **If No, please go directly to Question H.2**

**H.1.1: If Yes, did you seek health professional advice for this?**

Yes No

**H.1.2: For both ‘Yes’ and ‘No’ answers, did you receive advice from any other source?** *(Please circle all that apply)*

1. Radio/TV
2. Newspaper
3. Internet chat forum or blog
4. Pregnancy or parenting website
5. Phone app
6. Family and friends
7. Childbirth educator
8. Book or pamphlet

99 Other (Please specify) ____________________________________________________________

**H.2:** **In the last two weeks* did the strength of your baby’s movements** *(* before your baby died)*

01 Increase

02 Decrease

03 Stay the same

99 Unsure

**H.3:** **During the last two weeks* did the frequency of your baby’s movements** *(* before your baby died)*

01 Increase

02 Decrease

03 Stay the same

99 Unsure

**H.4: During the last two weeks* did you notice any time that your baby was more vigorous than usual?**

*(*before your baby died)*

Yes No

**H.4.1: If Yes:** Once More than once Unsure

**H.5: During the last two weeks*, did you feel your baby having hiccups?** *(*before your baby died)*

*(If unsure, describe as regular jerking movements happening at 1-2 second intervals over a period of 1-5 minutes)*

Yes No Unsure

**H.5.1: If Yes**  Once Occasionally Daily Unsure

**H.6: During the last two weeks, did you feel uterine contractions (tightenings/pre-labour contractions/ Braxton Hicks contractions/ false labour) for longer than an hour?**

Yes No Unsure

***Cases:*** *Please advise participants that these next few fetal movement questions may be difficult for them. Reassure them that there are no right or wrong answers to any of the questions.*

**H.7 During the last two weeks* did you ever feel concerned about baby’s movements?** *(*before your baby died)*

Yes No**Please go directly to next page to Question H.8**

**H.7.1: If Yes, what were your MAIN concerns about your baby’s movements?**  . (*Please select* ***all*** *answers that apply)*

1. Baby moved less often
2. Baby didn’t move at their usual active time
3. Baby was quiet (inactive) for longer than usual
4. Baby’s movements were not as hard
5. Baby didn’t move when you pushed or prodded your belly
6. Baby didn’t move when you had a drink (water or other liquid)

99 Other (Please specify) _________________________________________________________

**H.7.2: At the time how would you rate your concern?**

01 Slightly concerned

02 Moderately concerned

03 Very concerned

**H.7.3: Did you have a check-up with a midwife or doctor that included CTG monitoring or scan for this concern?**

Yes **If Yes, please go directly to next page to Question H.8**

No If No, please continue….

**H.7.3.1 Cases Only: If Yes, was baby’s death diagnosed at this time?**

Yes No

**Both Cases AND Controls**

**H.7.3.2: If you did NOT have a check-up** (with a midwife or doctor that included CTG monitoring or scan) **for your concern, what was the MAIN reason why?**

*(Please circle* ***one*** *answer only)*

1. Baby’s movements returned to normal
2. Friend or family member said this was normal
3. Phoned health professional (e.g. hospital or midwife/doctor) who said this was normal
4. Previous advice from health professional who said this was normal
5. Other source said this was normal (Specify) ______________________________________________
6. You thought baby was sleeping
7. You didn’t want to bother anyone
8. You decided to wait until the next scheduled appointment

99 Other (Please specify)________________________________________________________________

**H.8: In the last two weeks* on average how many ‘busy times’ did your baby have in a day**? (*before your baby died)

*(Describe ‘busy times’ as a period where there is a group of movements, rather than single isolated movements*

*For some women these might be short 15-45 seconds, for others these might be prolonged and involve many movements for up to 20 minutes)*

1. 0
2. 1-3
3. 3-5
4. 5-7
5. 7-10
6. 10-20
7. 20-30
8. 30+

**H.8.1: In the last two weeks* on average how long did these ‘busy times’ last?** *(*before your baby died)*

- 1. Longer than before
  2. About as long as before
  3. Shorter than before

**H.9: During the last two weeks* did you do moderate exercise at any time** *(*before your baby died)*

Yes No

**J. Injury**

**J.1: Did you experience any physical injury at any time during your pregnancy?**

*(Please tick all the relevant answers)*

No injury **(If no injury, please go directly to next page - Family violence)**

Slips and falls

MVA

Blow to abdomen

Self-harm

Other non-accidental

Other accidental

**J.1.1: If yes, did you see a health professional about this?**

###### Yes No

**J.1.2 If yes, was this in the last two weeks of your pregnancy?**

Yes No

**J.1.2.1: If Yes, please describe the physical injury**____________________________________

______________________________________________________________________________

______________________________________________________________________________

______________________________________________________________________________

______________________________________________________________________________

**K. Family violence**

*These questions must be asked only if the woman is on her own*

**Woman not on her own?**  *(If not on her own, go directly to next page for Cases or directly to*

*Clinical Data Collection for Controls)*

**Woman on her own but declined to answer?** *(If declined, go directly to next page for Cases or directly to*

*Clinical Data Collection for Controls)*

**Family violence questions not asked for other reason (please specify)** ___________________________________________________________________________________________

___________________________________________________________________________________________

___________________________________________________________________________________________

**If the woman is on her own and did not decline to answer the family violence questions:**

**In the past year…**

**K.1: Have you been hurt or frightened by someone close to you?** Yes No

**K.2: Have you felt controlled or always criticized in your relationship?** Yes No

**K.3: Have you been made to do anything sexual that you did not want to do?** Yes No

*Please refer to your local DHB protocol if there is a positive family violence screen*

**L. Other: Cases Only** Controls go directly to Clinical Data Collection

**L.1: I would finally like to ask you, what was the first reason that you thought something was wrong with your pregnancy or that your baby was dying/had died?**

*(Please circle one answer only)*

1. I felt a reduction of kicks/movements
2. I felt kicks/movements stop
3. I felt abdominal pain
4. I had vaginal bleeding/hemorrhage
5. I had discharge of amniotic fluid/the membranes ruptured
6. I had a "feeling that something was wrong", but cannot specify
7. I had a trauma (involved in a physical accident)
8. I had other symptoms (specify below if possible)
9. I was told at an appointment for prenatal care
10. I was told when I was admitted for labour
11. I was told during labour
12. It was not discovered before the baby was born

99 I do not remember/know

Comments: ______________________________________________________________________

L.2: When do you think that your baby died? _____/______/______ (DD/MM/YYYY)

L.3: What time of day do you think that your baby died? (Please circle one answer only)

1. During the night
2. During a daytime nap
3. In the morning
4. In the afternoon
5. In the evening

99 Not sure

**L.4: What was the reason you saw a health practitioner at the time that baby was found to have died?**

*(Please circle one answer only)*

1. Routine scheduled pregnancy visit
2. Routine scan
3. Decreased baby movements
4. In labour
5. In hospital
6. Vaginal bleeding
7. Rupture of membranes
8. Unwell

99 Other (specify) ________________________________________________________________

00 Not recorded/unknown

**L.5: Were you asked if you would like a postmortem for your baby?** Yes No

**L.5.1: If yes, did you choose to have a post mortem?** Yes No

**L.5.1.1: If no, what was the main reason you decided against a postmortem?**

*(Please circle one answer only)*

1. We already knew why baby had died
2. It would not bring baby back
3. Did not want baby to be taken away
4. Did not want baby to be cut
5. Wanted to bury baby as quickly as possible

99 Other (specify) _______________________________________________

**L.6: Would you make the same decision about the postmortem now?** Yes No

Is there anything else that you think might be important?

___________________________________________________________________________________________

___________________________________________________________________________________________

___________________________________________________________________________________________

___________________________________________________________________________________________

___________________________________________________________________________________________

___________________________________________________________________________________________

___________________________________________________________________________________________

___________________________________________________________________________________________

___________________________________________________________________________________________

Have you been told anything about why your baby may have died? Yes No

If Yes, what were you told? ___________________________________________________________________

___________________________________________________________________________________________

___________________________________________________________________________________________

___________________________________________________________________________________________

“Thank you for answering these questions, your answers are very important in helping us understand more about stillbirth.”

“The final part of this interview is the clinical data collection from your antenatal record “

| Study Number | Multi-Centre Stillbirth Study | Date |
| --- | --- | --- |
| __ __ __ __ __ __ | **Clinical Data Collection** | ____/____/____ |

Cases and Controls

**This data is to be collected from the antenatal record at time of interview if possible**

***Reminder to record the woman’s height and weight today (on page 1)***

**If the woman does not have her antenatal record available please obtain this clinical data from her LMC or hospital records**

1. **Current pregnancy**

**1.1:** **EDD by LMP**  _____/_____/_____ (DD/MM/YYYY) EDD not known

**1.2:** **EDD by USS**  _____/_____/_____ (DD/MM/YYYY) USS not done

**Gestation at first USS** ______ (weeks)

**1.3**: **Best agreed EDD**

(See protocol) _____/_____/_____ (DD/MM/YYYY)

**1.4:** **Height recorded in notes** ____________ (cms) Not recorded

**1.5: First weight in pregnancy**  __________ (kgs) Not recorded

**1.5.1**: **Gestation at first weight** __________ (weeks) NA

**1.5.2:** **Last weight** (prior to interview) __________ (kgs) Not recorded

**1.5.2.1: Gestation at last weight:** __________ (weeks) NA

**Antenatal care**

**1.6: Date of first visit with lead maternity carer (LMC)?** _____/_____/_____ (DD/MM/YYYY)

**1.7: Estimated gestational age at first visit with LMC?** ______/_______ (weeks/days)

**1.8: Initial LMC type?** (Who provided antenatal care)

*(Please circle one answer only)*

- 1. Hospital team midwife (continuity of care)
  2. Self-employed midwife
  3. Hospital DHB midwife (core midwives in DHB clinics and hospitals)
  4. Private obstetrician
  5. GP (circle only if GP provides both antenatal **and** birth care - otherwise record as 06 “Shared care”)
  6. Shared care (please specify both parties) ________________________________________________

1. Other (please specify) _______________________________________________________________

**1.9: Referral to obstetric/medical specialist?** Yes No

**1.9.1: If Yes, why?**

*(Please circle one answer only)*

01 Pre-existing condition

02 Complication of pregnancy

03 Maternal request

99 Other (please specify) ________________________________________________________

**1.9.2: Transfer of care?** Yes No

**1.10: Booked place of birth?**

*(Please circle one answer only)*

01 Tertiary hospital (Auckland City, Middlemore, Waikato, Wellington Women’s, Christchurch Women’s)

02 Secondary hospital

03 Primary birthing unit

04 Home

99 Other (please specify) _______________________________________________________________

**1.11: Number of antenatal visits in 1^st^ trimester?** **_________** (*from antenatal records from woman or LMC)*

**1.12: Number of antenatal visits in 2^nd^ trimester?** **________** (*from antenatal records from woman or LMC)*

**1.13: Number of antenatal visits in 3^rd^ trimester?** **_________** *(from antenatal records from woman or LMC)*

**No antenatal record available?**  (Please specify reason) _________________________________________

___________________________________________________________________________________________

**2. Screening and management this pregnancy:** Cases and Controls

**2.1:** **Ultrasound this pregnancy?** *(Please tick all the relevant answers)*

First trimester scan

Scan at ≤ 20/40

Doppler studies

Growth scan

None

**2.2: Medical conditions this pregnancy?** *(Please tick all the relevant answers)*

No medical conditions in pregnancy

Anaemia Other autoimmune

Asthma Renal disease

Cervix surgery Rheumatic heart

Depression Major psychiatric disorder (other than depression)

Epilepsy Systemic lupus erythematosus

Heart condition - congenital Thalassaemia trait

Heart condition – rheumatic Thrombophilia

Hyperthyroid Urinary tract infection

Hypothyroid Uterine abnormality

Inflammatory bowel Venous thromboembolism

Laparotomy Other (specify): ______________________________________

**2.3: Blood pressure at booking?** Systolic ________ Diastolic ________

**2.4: Last blood pressure: prior to interview *(controls)* or when baby last known to be alive *(cases)*?**

Systolic _______ Diastolic ________

**2.4.1: Gestation at last blood pressure?** _______ (weeks)

**2.5: Was a customised growth chart used this pregnancy?**

Yes No Don’t know

**2.5.1: If Yes, gestation first used?** _________ (weeks)

**2.6: Was fetal growth restriction clinically suspected this pregnancy?**

Yes No  (**If No, please go directly to Q 2.7 preterm labour)**

- - 1. **If Yes, gestation first suspected?** _________ (weeks)

**2.6.2: Was a growth scan done?** Yes No (**If No, please go to Q 2.7 preterm labour)**

**2.6.3: Was there evidence on the growth scan of fetal growth restriction this pregnancy?**

Yes ac <10%

Yes EFW <10%

No (**If No, please go directly to Q 2.7 preterm labour)**

**2.6.4: If Yes, what was management?** *(Please tick all the relevant answers)*

No change

Increased antenatal visits

Serial CTG’s

Ultrasound scan

Dopplers

Admitted

Delivered

Other (specify): ________________________________________________________

**2.7: Admitted with threatened preterm labour this pregnancy?**

Yes No Don’t know

**3. Laboratory Results:** Cases and Controls

**3.1: Blood group?**

1. A Pos
2. B Pos
3. AB Pos
4. O Pos
5. A Neg
6. B Neg
7. AB Neg
8. O Neg

99 Not known

**3.2: Hep B status?** Pos Neg Not tested

**3.3: Polycose performed?** Yes No  (Please fill in results and gestation)

**If Yes,** **please fill in results and gestation** **in weeks at time of test**

Result _________ Gestation_________ (wks)

Result **_________** Gestation_________ (wks)

**3.4: HbA1c performed?** Yes No

**3.4.1: If Yes**: Result **_________** Gestation **_________** (wks)

**3.5: GTT performed?** Yes No

**3.5.1: If Yes**: Fasting**_______**  1 hour**_______**  2 hour**_______** Gestation**_______** (wks)

Fasting**_______**  1 hour**_______**  2 hour**_______** Gestation**_______** (wks)

*“Thank you very much. Your interview is now complete.”*

Checklist:

Consent form is completed and attached

Height and weight measured and recorded on front page

Feedback form/stamped addressed envelope provided

Koha given

NHI recorded and attached to interview form

**4. Baby:** Cases and Controls

This data is to be collected from the hospital records or LMC

**4.1: Baby date of birth?** _____/_____/_____ (DD/MM/YYYY)

**4.2: Place of birth?**

01 Tertiary/Secondary hospital

02 Primary birthing unit

03 Home

99 Other (please specify) _______________________________________________________________

**4.3: Birth weight in grams?** **____________** (gms)

**4.4: Best estimate of gestation at birth *(for controls)* or at diagnosis of stillbirth *(for cases):***

______/_______ (weeks/days)

**4.5: Sex of baby?**

01 Male

02 Female

**Control Baby Data**

Unless the birth is imminent, please don’t wait for this baby data before sending the interview form to the Study Coordinator. The baby data can be emailed to the Study Coordinator after the birth.

**5. Details of Stillbirth:** Cases Only

**5.1: Date of diagnosis of fetal death?** _____/_____/_____ (DD/MM/YYYY)

**5.2: Date of last consult prior to death where fetus confirmed alive**? _____/_____/_____ (DD/MM/YYYY)

**5.3: New findings at last consult prior to diagnosis of fetal death?** *(Please tick all the relevant answers)*

No new findings

SGA

LGA

Hypertension

Oligohydramnios

Polyhydramnios

APH

Diabetes

Decreased fetal movements

Urinary tract infection

Other (specify): ______________________

**5.4: When did fetal death occur?**

01 Antepartum

02 Intrapartum

99 Unknown whether antepartum/intrapartum

**5.5: Postmortem?** Yes No

**5.5.1 If Yes, where was it done?** _______________________________________________________

*(Attach copy of results if available)*

**5.6: Placental pathology?** Yes No

**5.6.1: If Yes, where was it done?** ________________________________________________________

*(Attach copy of results if available)*
